# Beyond Criterion A: Employing a Three-Level Framework for Conceptualizing Youth Stressor Severity and Posttraumatic Stress Symptoms

**DOI:** 10.1007/s40653-026-00933-z

**Published:** 2026-06-30

**Authors:** Malena A. Nygaard, Stephanie M. Hernandez, Aaron Corn, Srividya Mehendale, Julie M. Petersen, Sarah Kate Bearman

**Affiliations:** 1 Department of Psychology, University of South Carolina, Columbia, United States; 2 Department of Psychology, Harvard University, Cambridge, United States; 3 The Ballmer Institute for Children’s Behavioral Health, University of Oregon, Eugene, United States

**Keywords:** Trauma, Potentially traumatic event, Criterion A, Posttraumatic stress, Youth

## Abstract

Children experiencing persistent posttraumatic stress symptoms can receive a diagnosis of post-traumatic stress disorder (PTSD), opening the gate to trauma-focused services. However, many youths are undiagnosed due to complex presentations or because their stressor exposure does not meet Criterion A (“Exposure to actual or threatened death, serious injury, or sexual violence…”; American Psychiatric Association, 2022, p. 302), despite impairing symptoms. The current study applies a novel, three-level framework based on increasingly strict stressor criterion to conceptualize stressor exposures and explore associated symptoms. We employed a convergent parallel, QUAL + quan design to analyze phone-administered baseline assessments of the UCLA PTSD Reaction Index for 438 caregiver-youth dyads seeking treatment as part of a larger randomized controlled trial in U.S. community mental health clinics. Through framework analysis of assessor notes, the following theme developed: *Some stressor types do not meet Criterion A but are reported as significant stressors*. In the quantitative results, fewer stressors met the increasingly strict severity criteria; yet there were cases of elevated symptoms for non-Criterion A stressors (*n* = 149). Findings support applying a three-level framework to youth trauma exposures.

According to the National Child Traumatic Stress Network, child trauma refers to a child witnessing or experiencing an event that poses a real or perceived threat to their life or well-being, or to the life and bodily integrity of someone close to the child (Elmore et al., 2023). Such events can include physical or sexual abuse, death or loss of a loved one, life-threatening illness of a caregiver, witnessing domestic violence, serious accidents, and community violence, among others (see NCTSN, n.d.). Exposure to potentially traumatic events is substantially common among children and adolescents (McLaughlin et al., 2013); globally, nearly two-thirds of youth report experiencing at least one potentially traumatic event before age 17, increasing their risk of experiencing multiple events overtime (Adams et al., 2016; Carlson et al., 2020; McLaughlin et al., 2013). When these potentially traumatic event(s) overwhelm a child’s ability to cope and the physiological and emotional reactions continue to persist after the event(s) has concluded, this can result in child traumatic stress that interferes with daily functioning (Elmore et al., 2023). Without treatment, repeated childhood exposure to traumatic events can affect the brain and nervous system (De Bellis & Zisk, 2014; Peverill et al., 2023), as well as impair social, emotional, behavioral, and academic functioning (e.g., Perfect et al., 2016).

## Post-Traumatic Stress Disorder

When child posttraumatic stress (PTS) symptoms are experienced simultaneously and persist over time, they can result in a formal diagnosis of post-traumatic stress disorder (PTSD). In the United States, the Diagnostic and Statistical Manual of Mental Disorders, 5th Edition, Text Revision (DSM-5-TR; American Psychiatric Association [APA], 2022) requires Criterion A, also referred to as the *stressor criterion*, be met for a diagnosis of PTSD:

Exposure to actual or threatened death, serious injury, or sexual violence in one (or more) of the following ways:

1. Directly experiencing the traumatic event(s).
2. Witnessing, in person, the event(s) as it occurred to others.
3. Learning that the traumatic event(s) occurred to a close family member or close friend. In cases of actual or threatened death of a family member or friend, the event(s) must have been violent or accidental.
4. Experiencing repeated or extreme exposure to aversive details of the traumatic event(s) (e.g., first responders collecting human remains; police officers repeatedly exposed to details of child abuse; APA, 2022, p. 302).

A diagnosis of PTSD also includes intrusion symptoms, avoidance of trauma reminders, negative changes in cognition or mood, and changes in arousal and reactivity that last more than one month, cause clinically significant distress/impairment, and are not attributable to another medical condition or substance use. In children 6 years of age or younger, PTS symptoms can be observed through play reenactments of the trauma, nightmares that may not be closely linked to the event, and extreme behavioral problems or temper tantrums (APA, 2022). Despite these seemingly clear definitions of childhood trauma and PTS, there has been further debate.

## The Stressor Criterion Debate

Since the addition of PTSD to the *DSM-III* (APA, 1980), the debate around the use of Criterion A in determining the type of event(s) that can be considered traumatic and elicit PTS symptoms has been ongoing (Gradus & Galea, 2022; Marx et al., 2023; Miller et al., 2014; Mol et al., 2018). Marx and colleagues (2023) outlined the debate about Criterion A and described the following four common stances amongst PTSD scholars: “(a) expanding Criterion A such that more events qualify, (b) narrowing Criterion A such that fewer events qualify, (c) eliminating Criterion A completely, or (d) leaving Criterion A in its present form” (p. 5). The *DSM-5-TR* (APA, 2022) clarified how subjective experiences are necessary for determining whether a medical event(s) meets Criterion A. With this debate in mind, the NCTSN indicates two-thirds of youth in the United States are exposed to a traumatic event before age 16 (Copeland et al., 2007; Elmore et al., 2023); however, only 4.7% of U.S. adolescents meet lifetime criteria for PTSD, perhaps due to restrictions related to criterion A (Alisic et al., 2018). Despite differences in defining stressor severity, more research is needed to examine if a variety of stressors (including those that do not meet Criterion A as currently defined) have the ability to evoke PTSD symptoms (Gradus & Galea, 2022; Mol et al., 2018; Weathers & Keane, 2007).

## A Three-Level Framework for Conceptualizing Youth Trauma Exposure Severity

To assuage differences in defining and classifying childhood trauma exposures, we propose a three-level framework to conceptualize youth trauma exposure severity (see Table 1). First, the current definition of Criterion A can be applied to stressors (Level A) that involve “exposure to actual or threatened death, serious injury, or sexual violence” (APA, 2022, p. 302). On the opposite end of the severity spectrum is when a youth or caregiver self-endorses a stressful event as “trauma” (Level C). Couched between the two are trauma exposures as defined by the NCTSN (2024). These Level B stressful events can include Level C events, but do not reach the required threshold to be considered Criterion A (Level A) events. By applying a three-level severity framework to youth trauma exposures, we can clarify how stressor severity is associated with PTS symptoms and aim to resolve concerns raised in the Criterion A debate.

**Table 1:** Analytic framework

Code	Definition	Examples
Criterion A Trauma Exposure (Level A)	“Exposure to actual or threatened death, serious injury, or sexual violence in one (or more) of the following ways: − Directly experiencing the traumatic event(s). − Witnessing, in person, the event(s) as it occurred to others. − Learning that the traumatic event(s) occurred to a close family member or close friend. In cases of actual or threatened death of a family member or friend, the event(s) must have been violent or accidental. − Experiencing repeated or extreme exposure to aversive details of the traumatic event(s) (e.g., first responders collecting human remains; police officers repeatedly exposed to details of child abuse)” (American Psychiatric Association [APA], 2022). “In children 6 years and younger, exposure to actual or threatened death, serious injury, or sexual violence in one (or more) of the following ways: 1. Directly experiencing the traumatic event(s). 2. Witnessing, in person, the event(s) as it occurred to others, especially primary caregivers. 3. Learning that the traumatic event(s) occurred to a parent or caregiving figure” (APA, 2022).	• Exposure to war as a combatant or civilian • Actual or threatened physical assault • Being kidnapped • Natural or human-made disasters • Severe motor vehicle accidents • Actual or threatened sexual violence • Bullying when there is a credible threat of serious harm or sexual violence • Life-threatening medical emergencies • Witnessed serious injury • Learning about violent or accident event that occurred to close relative/friend See DSM-5-TR for more examples.
Trauma Exposure (Level B)	A “frightening, dangerous, or violent event that poses a threat to a child’s life or bodily integrity” and overwhelms their ability to cope is trauma (National Child Traumatic Stress Network [NCTSN], 2024, para. 1). “When a child feels intensely threatened by an event he or she is involved in or witnesses,” this event is called a trauma (NCTSN, n.d., para. 1), and was coded as Level B whether or not it met threshold for Level A. The following stressor types required further clarification in coding. *** Bereavement***: Given the UCLA PTSD RI-5 asks if “someone you really cared about ever died,” this person was presumed to be close to the young person. Therefore, unless the participant indicated they were not close to the person, this stressor type was coded as Level B, if it did not meet for Criterion A. NCTSN (n.d., p. 1) states that “a child may have a traumatic reaction after a death that was sudden and unexpected (e.g., through violence or an accident) or a death that was anticipated (e.g., due to illness).” Thus, even deaths by “natural causes” were coded as Level B. *** Bullying***: Any report of bullying (physical, verbal, or social) was coded as Level B if there was an indication that it had been repeated more than one time. The NCTSN (n.d., para. 1) defines bullying as “a deliberate and unsolicited action that occurs with the intent of inflicting social, emotional, physical, and/or psychological harm to someone who often is perceived as being less powerful. Bullying typically happens repeatedly and is a form of aggression and harassment that prevents someone from enjoying a safe, stress-free living, learning, or working environment… Bullying can be physical (hitting, tripping, kicking, etc.), verbal (name calling, teasing, taunting, threatening, and sexual comments), and social (spreading rumors, embarrassing someone in public, being purposefully exclusive).” *** Domestic Violence***: Intimate partner violence occurs “when an individual purposely causes harm or threatens the risk of harm to any past or current partner or spouse” (NCTSN, n.d., para. 1). Children may “hear one parent threaten or demean the other, or see a parent who is angry or afraid. They may see or hear one parent physically hurt the other and cause injuries or destroy property” (NCTSN, 2014, para. 2). Any reports of physical or verbal violence between caregivers with some level of detail were coded as Level B. *** Illness/medical trauma***: Posttraumatic stress from medical trauma is “often more related to their [the child’s] subjective experience of the medical event rather than its objective severity” (NCTSN, n.d., para. 1). However, these events typically include “pain, injury, serious illness, medical procedures, and invasive or frightening treatment experiences” (NCTSN, n.d., para. 1). We coded an event as Level B when a youth went to the hospital, got a procedure, and it was described as distressing. *** Separation***: Separation from caregiver (inclusive of foster care) was coded as Level B when in a negative context or described as undesired. Divorce as a mechanism of separation from caregiver was included when described in detail with no access to one caregiver. The NCTSN (2016, para. 2) describes traumatic separation as: “sudden, unexpected, and prolonged…Situations in which a potentially traumatic separation from the caregiver can occur include: Parental incarceration, Immigration, Parental deportation, Parental military deployment, Termination of parental rights.” Threatened separation was not coded. *** Psychological maltreatment/emotional abuse***: The American Professional Society on the Abuse of Children (APSAC, 2002) defined emotional abuse as “a repeated pattern of caregiver behavior or a serious incident(s) that convey to children that they are worthless, flawed, unloved, unwanted, endangered, or only of value in meeting another’s needs” (p. 81). Therefore, this stressor type was coded as Level B when described as a recurring pattern.	• Physical, sexual, or psychological abuse and neglect (including trafficking) • Natural and technological disasters • Family or community violence • Terrorism, mass violence, and school shootings • Discrimination, prejudice, and racism • Sudden or violent loss of a loved one • Substance use disorder (personal or familial) • Traumatic separation (including as part of an immigration journey or incarceration) • Refugee and war experiences (including torture) • Serious accidents or life-threatening illness • Military family-related stressors (e.g., deployment, parental loss or injury) • Bullying (NCTSN, n.d.)
Self-Endorsed Trauma (Level C)	Participants endorsing “Yes” to an item on the Trauma/Loss History Screening Questions on the UCLA PTSD Reaction Index for Children/Adolescents was coded as Level C. The stressor type was recorded.	• Serious Accidental Injury • Illness/Medical Trauma • Community Violence • Domestic Violence • School Violence/Emergency • Physical Assault • Disaster • Sexual Abuse • Physical Abuse • Neglect • Psychological Maltreatment/ Emotional Abuse • Impaired Caregiver • Sexual Assault/Rape • Kidnapping/ Abduction • Terrorism • Bereavement • Separation • War/Political Violence • Forced Displacement • Trafficking/Sexual Exploitation • Bullying • Attempted Suicide • Witnessed Suicide

## Study Purpose

The purpose of this study is to explore (1) how potentially traumatic events (or stressors) are defined and classified and (2) the relationship between stressor severity level and PTS symptoms in a sample of youths and families who have sought treatment in community mental health clinics in the United States. We propose and apply a novel, three-level framework for conceptualizing the severity of trauma exposure, as operationalized through qualitative coding of detailed stressor descriptions from both youth and caregiver reports. Building on the ongoing debate over Criterion A as outlined by Marx et al. (2023), and responding to the call for research that empirically examines a wide array of stressful events (e.g., Gradus & Galea, 2022; Mol et al., 2018; Weathers & Keane, 2007), this study systematically explores stressor types, severity levels, and PTS symptoms in a diverse, treatment-seeking sample of youths from community clinics. By investigating how different definitions and classifications of stressor type relate to symptom elevations, this study seeks to inform clinical assessment and treatment, contributing to greater clarity regarding which kinds of stressors are more likely to meet diagnostic criteria for PTSD among youths.

## Method

### Study Design

This study is a secondary analysis of data collected as part of a randomized controlled effectiveness trial (RCET) of *The FIRST Program for Behavioral and Emotional Problems* (Weisz & Bearman, 2020) in community mental health clinics in Massachusetts and Texas. FIRST is a transdiagnostic mental health treatment that applies empirically supported principles to address youth anxiety, depression, trauma, and misbehavior. See Bailin et al. (2023) for a detailed description of the study protocol. For this study, we utilized a convergent parallel, QUAL + quan design (Creswell & Plano Clark, 2018).

### Recruitment and Participants

Baseline data from the RCET was collected by trained assessors who were naïve to youths’ treatment conditions (Bailin et al., 2023). Although the RCET utilized many measures collected at various time points (baseline, during treatment, quarterly, and post-treatment), our current study only includes data collected at baseline with the UCLA PTSD Reaction Index for *DSM-5* (RI-5; Pynoos & Steinberg, 2015). To be included in this study’s data analysis, at least one caregiver or youth participant needed to complete the RI-5 from September 2021 to March 2025.

The study included unique caregiver-youth dyads, represented by 438 total IDs. Participant information was primarily provided by caregivers (*n* = 220, 50.22% of total entries) and youths (*n* = 218, 49.77% of total entries). Demographic characteristics for participants with available data are detailed in Table 2. The average youth participant age was 11.25 (SD = 2.50) and caregivers commonly described their youth participants as White (*n* = 103, 46.8%), Black or African American (*n* = 55, 25%), Hispanic/Latinx (*n* = 101, 46.1%), and males (*n* = 112, 51.1%).

**Table 2:** Youth demographics reported by caregiver

Descriptor	Count (%)
	*n* = 220
Sex	
Female Male Not listed	106 (48.2) 112 (50.9) 2 (0.9)
Race	
Asian Black or African American More than one race Not listed Unknown or not reported White	8 (3.6) 55 (25.0) 24 (10.9) 26 (11.9) 4 (1.8) 103 (46.8)
Ethnicity	
Hispanic/Latinx Not Hispanic/Latinx Unknown/Unreported	101 (46.1) 117 (53.4) 1 (0.5)
Household income	
Less than $5,000 $5,000—$11,999 $12,000—$ 15,999 $16,000—$24,999 $25,000—$34,999 $35,000—$49,999 $50,000—$74,999 $75,000—$99,999 $100,000—$124,999 $125,000—$149,999 $150,000—$174,999 $175,000—$199,999 $200,000—and greater Prefer not to answer Not reported	9 (4.1) 5 (2.3) 10 (4.6) 27 (12.4) 34 (15.6) 35 (16.1) 22 (10.1) 14 (6.4) 15 (6.9) 6 (2.8) 5 (2.3) 5 (2.3) 19 (8.7) 12 (5.5) 1 (0.4)

### Study Measures and Data

#### Demographics

Caregivers reported youth age, race, gender, ethnicity, and their household income.

#### UCLA Child/Adolescent PTSD Reaction Index for DSM-5 (RI-5; Pynoos & Steinberg, 2015)

The RI-5 is a widely used, clinician-administered semi-structured interview that assesses PTS reactions and predicts PTSD diagnostic status in children and adolescents aged 7 to 18 who have been exposed to trauma (Kaplow et al., 2020). The assessment is typically completed in 20 to 30 minutes (Steinberg et al., 2004). The measure consists of six sections: (1) the *Trauma History Profile* assesses exposure to 23 types of traumatic events (e.g., serious accidental injury, bereavement, etc.); (2) the *Trauma Details* section assesses for specific trauma details (e.g., description of the traumatic event/s endorsed, age/s at which they occurred); (3) the *PTSD Symptom Scale* and (4) the *Frequency Rating Sheet* identify the number of days in the past month that the child/adolescent experienced each PTSD symptom (0 = no days to 4 = most days); (5) the *Distress and Impairment in Functioning* section rates whether symptoms cause clinically significant distress in various areas of the child’s life (e.g., home, school, with peers, etc.); and (6) the *Reaction Index Score Sheet* calculates whether *DSM-5* criteria for PTSD and dissociative subtype are met (Kaplow et al., 2020). Total RI-5 scores range from 0 to 80, with a clinical cut-off of greater than or equal to 35. Subscale scores for each criterion of the PTSD diagnosis can be calculated: re-experiencing (ranges from 0 to 20), avoidance (0–8), alterations in cognition/mood (0–28), alterations in arousal/reactivity (0–24). The RI-5 exhibits good internal consistency, criterion-referenced validity, and diagnostic accuracy in predicting PTSD diagnoses (Kaplow et al., 2020). In the present study, assessor notes from Section 2 of the RI-5 were treated as qualitative data, whereas scores from Sections 3–6 were treated as quantitative data.

### Data Analysis

#### Positionality

Our qualitative data analysis team consisted of one postdoctoral researcher with training in school psychology and trauma treatment and three postbaccalaureate research assistants. Two faculty members with expertise in evidence-based practices for youth mental health and an additional postdoctoral scholar with experience as a clinical psychologist served as auditors. Members identify as Indian, Mexican, and White, five identify as women and two identify as men, six live and work in the United States (e.g., Northeast, Pacific Northwest, Pacific states) and one lives and works in India. Through reflexivity journaling, analysis of analytic memos, and team discussion, we align with critical realism as our ontological and epistemological underpinning (Larkin et al., 2006) such that we believe context shapes interpretations and experiences of events, and in this case, stressful events. Throughout the qualitative data analysis process, we discussed how our positionality could shape our coding, interpretation, and analysis of the data, in alignment with recommendations from the field (Braun & Clarke, 2022; Nowell et al., 2017).

#### Qualitative Data Analysis

We used framework analysis, a type of thematic analysis intended to produce systematic results for applied policy research, to analyze the qualitative data (Gale et al., 2013; Ritchie et al., 2014). Unique to framework analysis, analyzed data is summarized into a matrix organized by rows (participant responses) and columns (themes; Frazer et al., 2023). Framework analysis consists of seven stages: transcription, familiarization, coding, creating an analytical framework, applying the analytical framework, organizing data into matrices, and interpretation (Gale et al., 2013).

The first four authors made up the coding team and completed Stages 2 through 7 as our dataset was already in a transcribed form (Stage 1). The coding team first familiarized themselves with the structure and contents of the dataset (Stage 2) by reading and discussing participants’ endorsed stressor types together and separately. Once familiarized, the authors deductively developed three codes (Stage 3) based on existing literature surrounding the classification of various stressor types by severity level: Criterion A stressor (Level A; APA, 2022), stressor exposure (Level B; NCTSN, 2024), and self-endorsed stressor exposure (Level C). The four coders completed two preliminary rounds of coding (30 IDs each round) to inform the development of the analytical framework (Stage 4) and reach intercoder reliability. The researchers met regularly throughout the coding process to discuss questions, disagreements, analytical memos, and to revise the analytic framework (Stages 4 and 5).

Intercoder reliability was calculated in Excel for the Level A and B codes applied to each stressor type endorsed using average percent agreement. Percent agreement was calculated rather than Cohen’s Kappa because code overlap was to be expected, and codes were not mutually exclusive (i.e., a stressor could be coded as both Level A and Level B; McHugh, 2012). For example, in an instance of domestic violence (“…[my dad] was threatening to burn the house down and threw [my] mom’s clothes in the oven…” [117 Y]), the youth experienced threatened death/serious injury; therefore, Level A (Criterion A) and Level B were coded. Coding Level A alone would be inaccurate because this stressor would also be considered a traumatic event according to the Level B criteria (NCTSN, 2024). Percent agreement met the minimum acceptable interrater agreement guidelines (McHugh, 2012) for each code (Level A and B) and overall reliability (i.e., average percent agreement across severity levels). For the first round, Level A average percent agreement was 85.83%, Level B was 83.78%, and the average total percent agreement was 84.80%. For round two, Level A average percent agreement was 83.19%, Level B was 80.87%, and total coder reliability was 82.03%.

Once reliability was met and the analytical framework was refined (see Table 1), two researchers per data segment coded the remaining dataset and Stages 5 through 7 were completed. To build trustworthiness, the coders organized data into matrices (Stage 6) and developed initial interpretations (Stage 7) individually. Each author abstracted encompassing themes and developed their own interpretations of the summarized data. The authors then met to discuss their candidate themes and interpretations. Each researcher developed similar themes to describe the data, thereby supporting the notion of successfully applying the analytic framework in a reproducible manner. Recurring research meetings occurred for peer debriefing to discuss questions during the coding process and reach consensus. The first theme is reported in this paper; participants are cited using study IDs and Y (for youth) or CG (for caregiver).

#### Quantitative Data Analysis

Quantitative data analysis took place in R with RStudio (R Core Team, 2024). Descriptive statistics were calculated to report means, standard deviations, sample sizes, and percentages of stressor types and severity levels for youths and caregivers in alignment with study aims.

### Mixed Methods Integration

Mixed methods analyses served the following functions: convergence (i.e., analyzing both quantitative and qualitative data provided triangulation surrounding the study purpose), complementarity (i.e., the quantitative results provide elaboration from the qualitative theme), and expansion (i.e., using the quantitative data to further explain and illustrate the qualitative theme; Palinkas et al., 2011). The mixed methods results are provided in Table 6 via a joint display (Guetterman et al., 2021). Ultimately, both data types were needed to comprehensively address our study aims.

## Results

### Qualitative Results

Through framework analysis of assessor notes from each item on the RI-5, the following theme developed: *Some stressor types commonly do not meet Criterion A level trauma for a PTSD diagnosis but are reported as significant stressors*.

### Theme: Stressor Types that Commonly Do Not Meet Criterion A

**Fig. 1:**
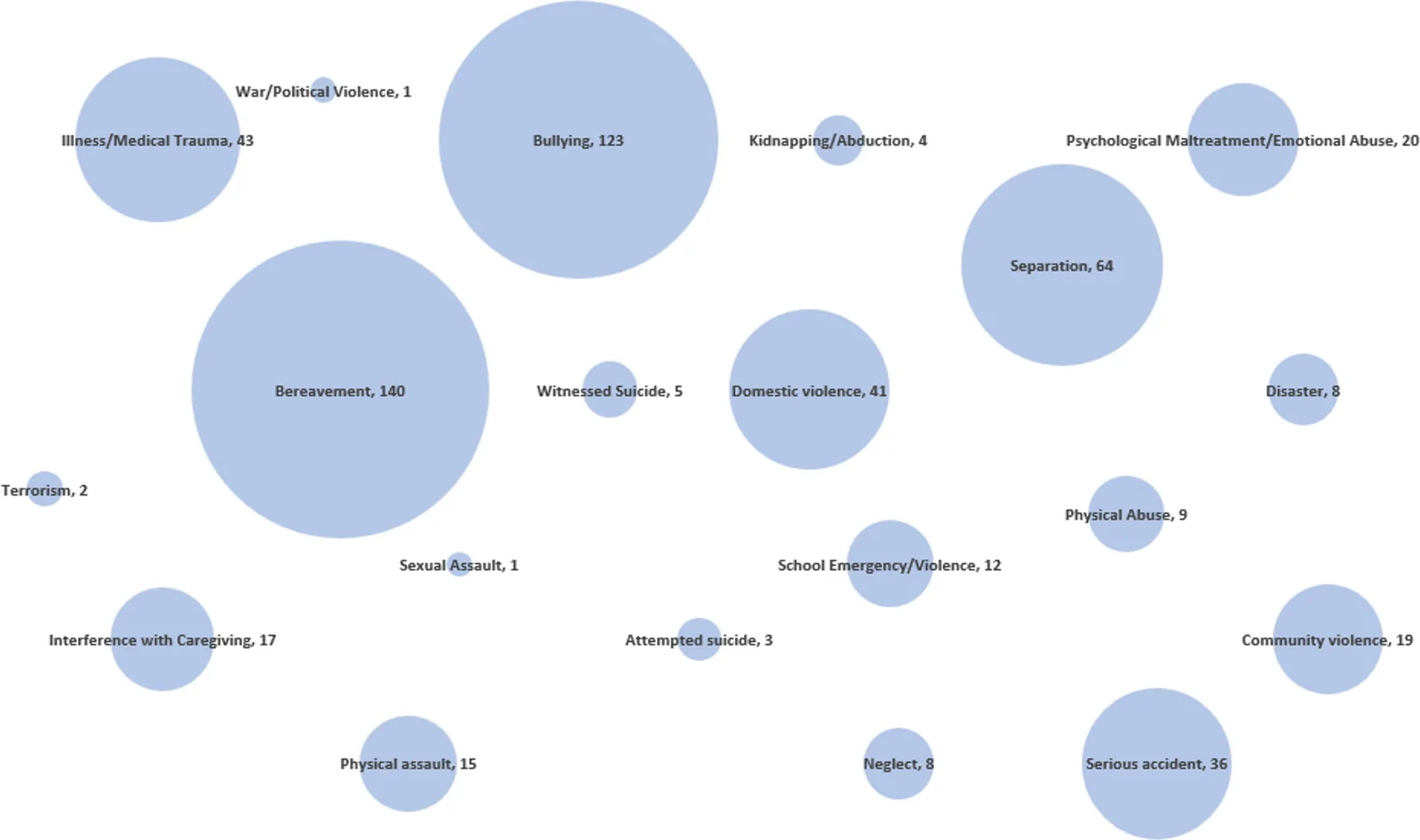
Bubble cloud of stressor types that commonly do not meet Criterion A. Note. The size of the bubble and digit after the stressor type label reflects the number of times the stressor type was categorized as Level B, and not Level A

*S*ome stressor types commonly do not meet Criterion A level trauma for a PTSD diagnosis but are reported as significant stressors. When qualitatively coding stressors for severity level (i.e., Level A, B, or C), many data extracts did not meet Criterion A (Level A) severity but were of greater severity than Level C (self-endorsed). Level B was the most commonly applied code and was the appropriate severity level for 20 of the 23 possible stressor types (e.g., bullying, community violence, witnessed suicide, etc.). Figure 1 demonstrates the pattern of which stressor types were commonly categorized as Level B stressors such that larger sized bubbles indicate a greater number of times in which that stressor type was categorized as Level B (and not Level A). The topmost stressor types meeting the medial severity level included: bereavement (*n* = 140), bullying (*n* = 123), separation (*n* = 64), illness/medical trauma (*n* = 43), domestic violence (*n* = 41), and serious accidental injury (*n* = 36).

#### Bereavement

For Criterion A, the loss of a close person must occur in a sudden/accidental or violent manner (APA, 2022). However, when youth in this sample lost someone close to them, it did not always occur in a violent way. For example, youths commonly experienced a grandparent’s death from cancer (e.g., 60 Y, 69 Y, 76 Y, 118 Y, 119 Y, 140 Y, 183 Y, 206 Y). Other close relatives died from illness (e.g., “he died of kidney failure” [94 CG and 49 CG]). Pet deaths were also reported but not included as Level B stressors (89 CG and 201 Y). Additionally, per assessor notes, some youths experienced potential PTS symptoms like avoidance and hyper-arousal during the assessment (e.g., “[221 Y] started hiding under the blankets while on the phone for this question”).

#### Bullying

For bullying to meet for Criterion A, the youth must have experienced actual or threatened death or serious injury (i.e., physical harm; APA, 2022). Youths and their caregivers reported experiencing repeated, deliberate, and unsolicited actions that had the intent to inflict social, emotional, or psychological harm, not only physical harm. Bullying was described to include (1) name calling: “People at school kept calling her names in Spanish like pendejo. Made child very mad” (56 Y); (2) verbal statements about physical appearances intended to cause harm: “fat” (17 CG, 43 CG, and 92 CG and Y); and (3) racism and discrimination: “She [the bully] expressed how she didn’t like Black people to her [the youth participant] and her friends…‘All Black people should die’…” (85 CG). This bullying was described as repetitive and spanned multiple environments: “Constant name calling and talking down. Curse words on social media” (64 CG). When bullying progressed to physical means, these events might not have met Criterion A level because the youth did not experience serious injury or threatened death: “There was also consistent physical pushing him around for a long time” (26 CG). However, lasting consequences were described: “Some girls at school had been picking on her, pushing her around, pulling her hair, and talking about her. [Caregiver] believes that this caused the self-harm” (23 CG). PTS symptoms, including avoidance and alterations in mood and cognition, were described by participants and linked to the bullying: “[155 Y] started doing homeschooling for the rest of this year due to these two bullies”; [244 Y] is “refusing to go to school… Every night and morning he gets very scared of the dark and doesn’t want to sleep”; “That’s what made me start thinking of killing myself because I’ve never had those thoughts…until what he was telling me” (241 Y).

#### Separation

Separation from caregivers was not categorized as Criterion A unless a threat of serious harm was present due to the separation (APA, 2022). Youths in this sample experienced separation from caregivers due to foster care (51 CG, 56 CG, 87 CG and Y, and 223 Y), incarceration (58 Y, 94 CG, 111 Y, and 172 CG and Y), and divorce (89 CG, 168 CG, and 233 CG), as well as other negative experiences (e.g., family violence, interference with caregiving [40 CG and Y, 53 CG, and 147 CG and Y]). Although reports of foster care did not meet Criterion A, the events that resulted in this change of home placement commonly did meet Criterion A (e.g., “Police report filed for family violence…so child was placed into foster care” [51 CG]). However, Criterion A did not capture the stressful experience of being separated from a caregiver when there was not a threat of serious injury or death to the child or caregiver:

When he was with his mom people came to pick him up from the house. He didn’t know why and neither did his mom. They took him and his brother to a foster care place. The next day, he left that place and went to three different houses before Grandma. Brother [was] adopted to a different family. (141 Y)

At times, family incarceration resulted in multiple instances of separation from caregivers: “Mom was in jail so Dad took care of him and then Dad went to jail, so [his] grandparents took care of him” (154 Y). Furthermore, the lack of information provided to youth related to caregiver separations was also demonstrated: “Youth is not aware of why father went to jail (all he knows is that dad did something bad)” (172 CG). Potential PTS symptoms like negative thoughts about the future were discussed: “Mom went to jail for…3 months… [Youth experiences] Lots of anxiety…about whether Mom would go to jail again” [73 CG]).

#### Illness/Medical Trauma

Stressors reported as illness/medical trauma met for Criterion A when there was a reported threat of serious injury or death (APA, 2022). Alternatively, medical stressors that did not involve serious threat of injury or death to the youth or a loved one but did involve going to the hospital and a procedure were commonly described. Non-death experiences with COVID-19 were mentioned (23 Y, 90 Y, 103 CG, and 125 CG):

Mom got COVID-19. She was very sick but did not have to go to the hospital. It lasted 1 to 2 weeks. [The child was] not able to see mom. Mom called to give updates. Child felt sad and scared…Mother recovered. (40 CG)

Parent surgeries were also concerning: “Mom has had 10 surgeries for various unrelated health issues. Paramedics had to come to the house often” (32 CG). Youth personal experiences with illness/medical stressors were related to chronic health disorders (e.g., “asthma” [80 Y and 134 Y] and “diagnosis of diabetes was scary for youth” [118 Y]). Participants described youth avoidance of trauma-related stimuli after the medical event: “for 4-months after…that really traumatized her, she refused to do shot[s]…” [111 CG]).

#### Domestic Violence

Domestic violence met for Criterion A when there was witnessed or experienced threat of serious injury or death to a youth’s caregiving figure (APA, 2022). In some instances of reported domestic violence, caregivers were unsure what the youth witnessed or knew about the situation: “Her biological father got into a fight with mom, pushed mom, so he was kicked out and arrested…Not sure how much she saw, she was in the other room…” (14 CG). Domestic violence was described to occur behind closed doors at times: “Child was home when Mom was getting attacked by Dad. Child did not see anything but heard Mom and Dad yelling at each other, but the door was closed.” (192 CG). Therefore, youth understanding of the event(s) varied, with some youths likely missing details to conceptualize the severity of the stressor (e.g., 175 Y). Other youths took an active role in responding to the domestic violence, although the severity of the event did not meet Criterion A: “Dad was really mad at mom. He grabbed her really hard and [the] child called the cops” (35 Y).

#### Serious Accident

Serious accidental injury typically refers to a serious vehicular accident, fall, dog bite, or near drowning experience in which someone was badly hurt or killed (Kaplow et al., 2020). When an accident results in serious injury or death, and the youth is made aware or experienced this, the stressor would meet for Criterion A (APA, 2022). However, in our sample, youths commonly experienced car accidents that did not result in serious injury or death (9 Y, 50 CG, and 130 CG) but could have been experienced as scary, stressful, or potentially traumatic. For example, one youth and their family “got T-boned by another vehicle, mother was driving and sibling was there…no injuries” (128 CG). Similarly, another youth “was in a car with…Mom and brother, and the car hit a pole or tree…Child was not severely hurt, nor was anyone else in the car” (199 CG). Youths were described as “very scared” (120 CG) and “crying uncontrollably” (130 CG) when their vehicles were hit. In somewhat more severe cases that still did not meet Criterion A, more extreme measures were taken to ensure safety: “they were coming from Mexico on a bus, and it crashed with an 18-wheeler, mom opened the emergency window and had to get her [the youth participant] and her nephews out” (137 CG). One of the most severe cases of serious accidental injury in our sample did not meet Criterion A because the youth was unaware of the extent of the accident:

Mom was driving a large SUV and it was raining and they hit another car. He [youth] was in the back seat and was pretty safe. The person in the other car did die but [youth participant] did not see that happen. He may have heard that the person died but has never mentioned it. (32 CG)

Avoidance PTS reactions to serious accidents were described: “Now when the child is in the car and she sees a big truck; she always tries to move to the middle seat” (151 CG).

### Quantitative Results

**Fig. 2:**
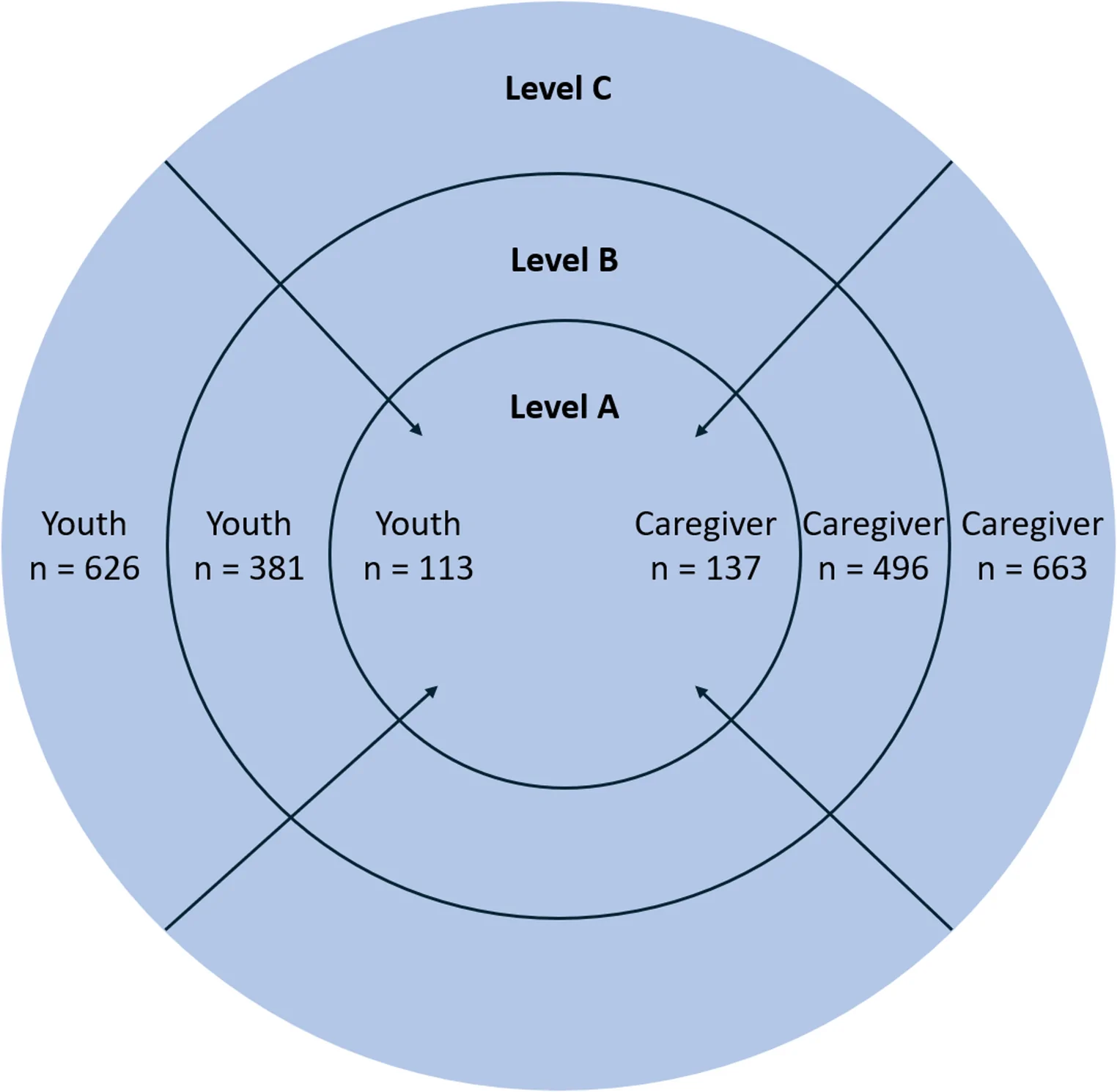
Frequency of stressors across severity levels

After qualitatively coding stressor severity levels, we examined caregiver and youth frequency counts as depicted in Table 3; Fig. 2. In total, 626–663 stressors were self-endorsed and met the Level C criteria based on youth and caregiver respective reports, 381–496 met Level B, and 113–137 met Level A, illustrating a pattern of decreasing stressors that met the increasingly strict criteria.

**Table 3:** The number of youth and caregiver reported stressors according to severity level

	Level A stressors			Level B stressors		Level C stressors	
	Youth (%)		Caregiver (%)	Youth (%)	Caregiver (%)	Youth (%)	Caregiver (%)
0 1 2 3 4 5 6 7 8 9 10 11 12 13 16		158 (72.48) 32 (14.68) 13 (5.96) 8 (3.67) 5 (2.29) 1 (0.46) 1 (0.46) 0 (0) 0 (0) 0 (0) 0 (0) 0 (0) 0 (0) 0 (0) 0 (0)	131 (59.55) 58 (26.36) 20 (9.09) 7 (3.18) 2 (0.91) 2 (0.91) 0 (0) 0 (0) 0 (0) 0 (0) 0 (0) 0 (0) 0 (0) 0 (0) 0 (0)	60 (27.52) 58 (26.61) 46 (21.10) 26 (11.93) 11 (5.05) 7 (3.21) 4 (1.83) 3 (1.38) 0 (0) 1 (0.46) 2 (0.92) 0 (0) 0 (0) 0 (0) 0 (0)	36 (16.36) 48 (21.82) 51 (23.18) 46 (20.91) 14 (6.36) 9 (4.09) 8 (3.64) 5 (2.27) 3 (2.36) 0 (0) 0 (0) 0 (0) 0 (0) 0 (0) 0 (0)	46 (21.1) 31 (14.22) 37 (16.97) 29 (13.3) 23 (10.55) 20 (9.17) 12 (5.5) 10 (4.59) 5 (2.29) 1 (0.46) 0 (0) 1 (0.46) 2 (0.92) 0 (0) 1 (0.46)	21 (9.55) 37 (16.82) 42 (19.09) 49 (22.27) 26 (11.82) 13 (5.91) 15 (6.82) 6 (2.73) 8 (3.64) 1 (0.45) 1 (0.45) 0 (0) 0 (0) 1 (0.45) 0 (0)

Regarding stressor types, bereavement (*n* = 217) was the topmost commonly endorsed stressor type followed by bullying (*n* = 186), illness/medical trauma (*n* = 160), serious accidental injury (*n* = 121), separation (*n* = 115), and domestic violence (*n* = 108). The least commonly endorsed stressor type was forced displacement (*n* = 0). Table 4 lists the stressor types endorsed by youths and caregivers in order of most to least commonly endorsed.

**Table 4:** Youth and caregiver combined stressor types endorsed

Stressor Type	Frequency Count
Bereavement	217
Bullying	186
Illness/medical trauma	160
Serious accidental injury	121
Separation	115
Domestic violence	108
Psychological maltreatment/emotional abuse	57
Impaired caregiver	50
Community violence	49
School violence/emergency	39
Neglect	37
Physical assault	36
Physical abuse	35
Disaster	29
Attempted suicide	21
Witnessed suicide	18
Sexual abuse	17
Sexual assault/rape	17
Kidnapping/abduction	12
Terrorism	2
War/political violence	2
Trafficking/sexual exploitation	2
Forced displacement	0

Mean total and criterion-specific RI-5 scores for youth and caregivers across severity levels are reported in Table 5. Youth and caregiver average RI-5 scores were slightly increased when at least one Level A stressor was endorsed (caregiver M = 28.4; youth M = 35.7) as compared to Level B and C stressors only (caregiver M = 25.1; youth M = 28.2). However, Level A stressors did not account for all of the scores that were above the clinical cutoff for PTSD symptoms, see Table 5. Of the 31 caregiver- and 46 youth-reporters who self-endorsed (Level C) stressors and had elevated PTSD symptoms, only 14 caregivers and 12 youth met Criterion A and had a probable PTSD diagnosis based on clinical scoring. Of note, those reporting at least one Level A stressor reported the highest re-experiencing (caregiver M = 7.0; youth M = 8.4) and alterations in cognition/mood scores (caregiver M = 9.9, youth M = 12.4).

**Table 5:** Posttraumatic stress symptoms according to stressor severity level

	Level A		Level B		Level C	
	Caregivers *n* = 65	Youth *n* = 47	Caregivers *n* = 119	Youth *n* = 115	Caregivers *n* = 127	Youth *n* = 124
RI-5 total scores, M(SD)	28.4 (17.1)	35.7 (19.8)	25.2 (17.4)	28.8 (19.8)	25.1 (17.2)	28.2 (19.8)
Elevated RI-5, n(%)	18 (20.22)	23 (38.33)	29 (15.76)	43 (27.22)	31 (15.58)	46 (26.74)
Probable PTSD diagnosis, n(%)	14 (15.73)	12 (20.0)	N/A	N/A	N/A	N/A
RI-5 Criteria Scores^1^ M(SD)						
Criterion B	7.0 (5.2)	8.4 (5.9)	5.8 (4.9)	6.6 (5.7)	5.8 (4.8)	6.4 (5.7)
Criterion C	3.3 (2.4)	4.8 (2.4)	3.0 (2.5)	3.6 (2.7)	3.0 (2.5)	3.5 (2.7)
Criterion D	9.9 (6.7)	12.4 (7.6)	8.7 (6.8)	9.8 (7.3)	8.7 (6.7)	9.6 (7.4)
Criterion E	8.3 (5.2)	10.2 (6.0)	7.6 (5.3)	8.9 (6.3)	7.7 (5.3)	8.9 (6.3)

Note. Some youth (*n* = 94) and caregiver (*n* = 93) RI-5 symptom scores are missing due to study procedural changes

^1^Criterion B: Re-experiencing, Criterion C: Avoidance, Criterion D: Negative alterations in cognition and mood, Criterion E: Alterations in arousal and reactivity

In addition to elevations of PTS total symptoms according to stressor severity level, Criterion B (re-experiencing), C (avoidance), D (negative alterations in cognition and mood), and/or E (alterations in arousal and reactivity) were elevated for the most commonly endorsed stressors. More specifically, 63% (*n* = 34) of 54 participants with *bereavement* as their primary stressor reported elevated re-experiencing (Criterion B) symptoms, 52% (*n* = 28) had elevated avoidance (Criterion C), and 43% (*n* = 23) had elevated alterations in cognition and mood (Criterion D) and changes in arousal and reactivity (Criterion E). Forty-seven participants endorsed *bullying* as their primary stressor: 70% (*n* = 33) met Criterion B, 47% (*n* = 22) Criterion C, 55% (*n* = 26) Criterion D, and 70% (*n* = 33) Criterion E. Of the 21 participants who endorsed *illness/medical trauma* as their primary stressor, 57% (*n* = 12) were elevated for Criterion B, 33% (*n* = 7) for Criterion C and E, and 24% (*n* = 5) for Criterion D. Of the 13 participants with *serious accidental injury* as their primary stressor, 46% (*n* = 6) were elevated for Criterion B and E, 38% (*n* = 5) for Criterion C, 31% (*n* = 4) for Criterion D. For 39 participants who endorsed *separation* as their primary stressor, 38% (*n* = 15) were elevated for Criterion B, 41% (*n* = 16) Criterion C, 31% (*n* = 12) Criterion D, and 51% (*n* = 20) Criterion E. Nineteen participants endorsed *domestic violence* as their primary stressor: 74% (*n* = 14) were elevated for Criterion B, 58% (*n* = 11) for Criterion C and E, and 42% (*n* = 8) Criterion D.

### Integration of Qualitative and Quantitative Findings

Table 6 illustrates the integration of the qualitative and quantitative findings via a side-by-side joint display.

**Table 6:** Side-by-side joint display of study findings

Qualitative Results	Quantitative Results	Combined Interpretation
Aim 1: Explore stressor types and severity levels		
Theme: Some stressor types commonly do not meet Criterion A level trauma for a PTSD diagnosis but are reported as significant stressors.	*Total stressors* Level A: *N* = 250 Level B: *N* = 877 Level C: *N* = 1,289 *Total stressor types* Bereavement: *n* = 217 Bullying: *n* = 186 Illness/medical: *n* = 160 Serious injury: *n* = 121 Separation: *n* = 115 Domestic violence: *n* = 108	The topmost commonly endorsed stressor types (quan) were also the stressor types that commonly did not meet for Criterion A but were reported as significant stressors and were coded as Level B (qual). The qualitative and quantitative results converged and indicate the following stressor types are prevalent but likely do not result in a diagnosis of PTSD due to stressor severity: Bereavement, bullying, domestic violence, illness/medical trauma, separation, and serious accidental injury.
Aim 2: Explore posttraumatic stress symptoms		
	*Elevated RI-5 symptoms* Level A: *n* = 41 Level B: *n* = 72 Level C: *n* = 77 *Probable PTSD diagnosis* *n* = 26	Although PTS symptoms may not be assessed if a Criterion A event is not endorsed and reported as the primary stressor, (quan) results suggest respondents reported clinically elevated PTS symptoms for Level B and C stressors (qual). Table 5 depicts average symptom scores across criterion.

## Discussion

In this study, we sought to explore stressor definitions and the relationship between stressor severity level and PTS symptoms, in response to the debate over Criterion A (Marx et al., 2023). We applied a novel, three-level framework (see Table 1) to RI-5 data collected as part of a U.S.-based RCET of a transdiagnostic mental health treatment tested in community mental health. Ultimately, our mixed method approach indicated that stressor severity does not do a very good job of identifying those with symptoms: we found 149 cases in which stressors did not meet Criterion A (and were coded as either Level B or C), but youth/caregivers rated elevated levels of PTS on the RI-5. Qualitative analysis suggested: *Some stressor types commonly do not meet Criterion A level trauma for a PTSD diagnosis but are reported as significant stressors.* Study findings suggest more attention must be paid to the assessment of PTS for youths who have experienced commonly overlooked stressors that do not meet Criterion A, within the context of other diagnoses that might also—or better—suit their elevated symptoms.

### Aim 1: Stressor Types and Severity Levels

Qualitative and quantitative study results converged such that the most commonly endorsed stressor types (see Table 4) did not meet Criterion A level trauma. Therefore, several frequently endorsed stressor types that *do not* meet Criterion A (but are associated with elevated symptoms) may require special attention in assessment and service planning. Study results suggest that current diagnostic guidance may miss some youths with PTS symptoms based on the current definition of Criterion A, particularly for youths who endorsed bereavement, bullying, domestic violence, illness/medical trauma, separation, and serious accidental injury. Notably, over half of participants who endorsed bereavement, bullying, illness/medical trauma, and domestic violence as their primary stressor reported clinically significant symptoms of re-experiencing (Criterion B). This gap in stressor severity and PTS symptoms is consistent with recent critiques of Criterion A and empirical work showing PTSD-like symptoms after non-Criterion-A events (Marx et al., 2023; Scheeringa et al., 2011). More specifically, there is growing evidence linking bereavement, bullying (including cyberbullying), domestic/intimate-partner violence exposures, illness/medical trauma, caregiver separation/placement instability, and serious accidental injury to PTS symptoms or other trauma-related concerns in youth (Idsoe et al., 2021; Kaplow et al., 2014).

### Aim 2: PTS Symptoms

In addition to paying special attention to specific stressor types that will likely not meet Criterion A, our study results suggest standard triage questions that terminate assessment when Criterion A is denied have the potential to overlook youths with clinically significant symptoms. Study participants regularly reported clinically elevated PTS symptoms for Level B and C stressors. Indeed, study results suggest youth with at least one Criterion A level stressor exposure had the highest levels of re-experiencing and alterations in cognition/mood scores. However, not all PTSD criterion were highest for this sub-sample, further underscoring the importance of assessing symptom scores even when Criterion A is not met. Taken together, based on study results and to identify the best fitting diagnosis (e.g., adjustment disorder, other specified trauma- and stressor-related disorder, unspecified trauma- and stressor-related disorder; APA, 2022), routine symptom screening (e.g., PTSD symptom checklists) should be administered regardless of whether youth endorse Criterion A items. Relying on responses to questions like “Did something really bad happen?” may not be adequate for identifying those with PTS symptoms warranting trauma-related treatment. The RI-5 and other measures have shown utility across a range of stressor exposures and can be used as symptom assessments to inform intervention, even when Criterion A is not endorsed (Scheeringa et al., 2011).

### Other Implications for Practice

Given our study results, which highlight discrepancies between stressor severity and PTS symptoms, a diagnosis-driven gate to care would likely exclude many symptomatic youths. Therefore, adopting symptom-based referral criteria or stepped-care models might better suit trauma types and symptoms, including symptoms that might be better categorized by a different disorder (i.e., not PTSD; Bailey et al., 2022). For youths with Level B or C exposures and elevated PTS symptoms, clinicians can consider: (a) brief trauma-focused interventions in school or other natural environments like primary care, (b) group or modular brief treatments, followed by, and stepped up to, (c) full trauma treatment when symptoms persist. This reduces unnecessary diagnostic gatekeeping while offering targeted support early (de Arellano et al., 2014). Notably, trauma interventions that incorporate trauma narration and gradual exposure to trauma reminders would likely be inappropriate for youth with lower severity levels of exposure without PTS symptoms such as re-experiencing and avoidance. In this case, clinicians can consider implementing interventions targeting symptoms rather than the stressor/memories.

Universal and school-based prevention/intervention are other avenues to consider for youths with Level B/C stressor exposures and PTS symptoms. For example, a school-based trauma-focused prevention program was co-facilitated by clinicians and classroom teachers in elementary school classrooms bringing trauma psychoeducation and resilience skills to the entire class (Nygaard et al., 2025). Because many Level B stressors can occur in school and peer contexts (bullying, separation effects manifesting in school performance), investing in universal trauma-sensitive supports, teacher training, and early intervention to reach youths who may never present to specialty care contexts is warranted (Plexousakis et al., 2019). At the same time, youths who experience emotional abuse, chronic neglect, or identity-based harassment frequently have both higher symptom burden and more barriers to care (e.g., stigma, provider scarcity, systemic bias; Asiedu & Baliki, 2025). Therefore, implementation strategies can include workforce training, culturally responsive outreach, and low-barrier delivery like telehealth and school-based service delivery (Asiedu & Baliki, 2025).

### Limitations

This study is not without its limitations. To start, assessor notes were not verbatim transcripts. Therefore, missing nuance or omitted details could have biased severity coding, a recognized limitation when phone or checklist assessments are used without audio transcripts. Future studies could consider audio-recorded assessments (with consent) or structured follow-ups to minimize information loss. There was also an assessment procedure change in the parent RCET during the course of data collection resulting in missing RI-5 symptom data for cases that did not meet Criterion A after a certain point. There are sampling/generalizability limitations because our study sample was drawn from community mental health clinics in two U.S. states. Larger, population-representative replication is needed to quantify the public-health implication of missed cases. Within our sample study exclusion criteria (youths with autism spectrum disorder, high suicidality, and/or who were in foster care or for whom birth parents had lost custody) may have systematically excluded some high-need youths. Future work can consider targeting these subgroups given known trauma–suicidality links (Hoover, 2015; Johnson, 2017). Finally, for the purposes of this study, we combined youth and caregiver reports in our results. Follow-up studies should distinguish between reporters and compare youth and caregiver reports.

## Conclusion

This study found that applying a three-level framework to conceptualize youth trauma exposure severity is a more comprehensive mechanism through which to categorize stressor severity than solely relying on Criterion A (or not). This paper responds to the debate over Criterion A (Marx et al., 2023) and the call to explore stressor severity and PTS symptoms. With the growing youth mental health concerns globally, we offer preliminary guidance to clinicians engaging in trauma screening and assessment.

## Data Availability

Data may be made available upon reasonable request and approval from the relevant institutions and IRBs; data are not currently available given pre-existing ethical restrictions from relevant institutions and IRBs.
